# Clinical outcomes of patients with *CIC*-rearranged sarcoma: a single institution retrospective analysis

**DOI:** 10.1007/s00432-024-05631-7

**Published:** 2024-03-04

**Authors:** Jacob Murphy, Erin E. Resch, Christopher Leland, Christian F. Meyer, Nicolas J. Llosa, John M. Gross, Christine A. Pratilas

**Affiliations:** 1 Johns Hopkins University School of Medicine, 733 N Broadway, Baltimore, MD 21205 USA; 2 Department of Oncology, The Sidney Kimmel Comprehensive Cancer Center, Johns Hopkins University School of Medicine, 1650 Orleans St, Baltimore, MD 21287 USA; 3 Department of Pathology, Johns Hopkins University School of Medicine, 401 N Broadway, Baltimore, MD 21231 USA; 4 Department of Orthopedic Surgery, Massachusetts General Hospital, Boston, USA

**Keywords:** Sarcoma, *CIC-*rearranged, Small round cell sarcoma

## Abstract

**Purpose:**

*CIC*-rearranged sarcomas represent a type of undifferentiated small round cell sarcoma (USRCS) characterized by poor survival, rapid development of chemotherapy resistance, and high rates of metastasis. We aim to contribute to the growing body of knowledge regarding diagnosis, treatment, clinical course, and outcomes for these patients.

**Methods:**

This case series investigates the clinical courses of ten patients with *CIC*-rearranged sarcoma treated at the Johns Hopkins Hospital from July 2014 through January 2024. Clinical data were retrospectively extracted from electronic medical records.

**Results:**

Patients ranged from 10 to 67 years of age at diagnosis, with seven patients presenting with localized disease and three with metastatic disease. Tumors originated from soft tissues of various anatomic locations. Mean overall survival (OS) was 22.1 months (10.6–52.2), and mean progression-free survival (PFS) was 16.7 months (5.3–52.2). Seven patients received intensive systemic therapy with an Ewing sarcoma-directed regimen or a soft tissue sarcoma-directed regimen. Three patients experienced prolonged disease-free survival without systemic treatment.

**Conclusion:**

Most patients in this case series demonstrated aggressive clinical courses consistent with those previously described in the literature, although we note a spectrum of clinical outcomes not previously reported. The diversity of clinical courses underscores the need for an improved understanding of individual tumor biology to enhance clinical decision-making and patient prognosis. Despite its limitations, this article broadens the spectrum of reported clinical outcomes, providing a valuable addition to the published literature on this rare cancer.

## Introduction

Sarcomas are an uncommon group of malignant connective tissue tumors, comprising less than 1% of adult malignancies and approximately 15% of childhood malignancies (HaDuong et al. 2015). Among these, undifferentiated small round cell sarcomas (USRCSs) comprise multiple subcategories, including the recently described *CIC*-rearranged sarcomas, sarcomas with *BCOR* alterations, and non-ETS fused sarcomas (such as NFATC2 and PATZ1 sarcomas) (Nagy 2020). Historically, these tumors were often categorized as belonging to the greater Ewing sarcoma family of tumors (ESFT) given the histologic appearance of a small round blue cell sarcoma; however, recent innovations in molecular and cytogenetic analysis have distinguished them as unique entities, which may assume different clinical behavior patterns. For instance, most *CIC*-rearranged sarcomas are diagnosed at a later age of onset (32 years) than Ewing sarcoma (Antonescu et al. 2017; Sankar and Lessnick 2011, Palmerini 2023). Furthermore, *CIC*-rearranged sarcomas typically arise within soft tissue, in contrast to Ewing sarcoma which more commonly arises from bone (Antonescu et al. 2017).

*CIC*-rearranged sarcomas most commonly harbor fusions involving the *DUX4* gene creating a chimeric fusion, *CIC::DUX4. * The *CIC::DUX4* fusion juxtaposes the DNA-binding domain of the Capicua (*CIC*) transcriptional repressor with the variable region of the double homeobox 4 (*DUX4*) transcription factor via a *t(4;19)(q35;q13)* translocation (Italiano et al. 2012; Rekhi et al. 2023; Satomi et al. 2022). This gene fusion removes the repressor function of *CIC* and instead promotes transcriptional activation of *CIC* downstream targets, such as the *PEA3* gene subclass of ETS family proteins including *ETV1, ETV4,* and *ETV5* (Choi et al. 2013; Graham et al. 2012; Italiano et al. 2012; Kawamura-Saito et al. 2006; Yoshida et al. 2016). However, other *CIC* rearrangements in USRCSs have been identified, such as *t(10;19)(q26;q13)* and *t(X;19)(q13;q13.3*), encoding the *CIC-FOXO4* gene fusion (Panagopoulos et al. 2023; Sugita et al. 2014; Italiano et al. 2012). These alternate *CIC* fusions result in a truncated CIC protein with a loss of critical DNA-binding capacity.

Reported outcomes for patients with *CIC*-rearranged sarcomas are generally associated with rapid development of chemotherapy resistance, high rates of metastasis, and poor overall survival (Antonescu et al. 2017; Connolly et al. 2022; Italiano et al. 2012; Mehdi et al. 2021; Yoshida et al. 2016). Most patients are treated with multimodal therapy—including combination chemotherapy, surgery, and/or radiation therapy—typically following established paradigms for the treatment of Ewing sarcoma or soft tissue sarcomas (STS). To date, there has been no consensus regimen or prospective randomized controlled clinical trials for the treatment of *CIC*-rearranged sarcomas, and there is no consensus on how to stratify these patients for treatment (Kinnaman et al. 2020; Mehdi et al. 2021; Palmerini et al. 2023). Herein, we aim to report our institutional and consultation experience regarding the diagnosis, treatment, and behavior of a cohort of *CIC*-rearranged sarcomas. We provide a retrospective review of ten patients, including three patients who achieved long-term survival without intensive Ewing sarcoma-like or STS-like chemotherapy.

## Methods

This retrospective study evaluated patients diagnosed with a *CIC*-rearranged sarcoma who were evaluated and/or treated at the Johns Hopkins Hospital (JHH) from July 2014 through January 2024. Patients were included if they had a diagnosis of *CIC*-rearranged sarcoma (all cases were re-reviewed by a bone and soft tissue pathology expert; JMG) and received treatment and/or diagnostic consultation at JHH.

Clinical data were retrospectively extracted from medical records including clinical course, radiologic, pathologic, and molecular genetic features, and treatment plans (*i.e.*, surgery, chemotherapy regimens, or radiotherapy regimens). Chemotherapy details included the combination of agents and time course of administration. Within regimens, dosages, length of cycles, and number of cycles administered were at the discretion of the treating physician. Specifics regarding ancillary study protocols (immunohistochemistry and/or molecular genetics) have been described previously (Wangsiricharoen et al. 2023; He et al. 2016; Italiano et al. 2012). Briefly, targeted next-generation sequencing (NGS) was performed on formalin-fixed paraffin-embedded (FFPE) tissue sections (*n* = 4) via the targeted NGS platform through FoundationOne Heme (Foundation Medicine, Inc.) (He et al. 2016). Five cases were evaluated via fluorescence in situ hybridization (FISH) performed on interphase nuclei from FFPE tissue using bacterial artificial chromosome (BAC) clones for *CIC* and/or *DUX4* rearrangement as previously described (Italiano et al. 2012). Three cases underwent molecular analysis at the Johns Hopkins Hospital using a custom, in-house, NanoString technology-based fusion panel (Johns Hopkins Comprehensive Sarcoma Fusion Panel) which allows for the detection of over 200 specific gene fusions in a single reaction. This assay will only detect specific targeted fusions and may not detect rare fusions with unknown breakpoints (Wangsiricharoen et al. 2023).

Time to disease progression was defined as the time from pathologically confirmed diagnosis to the first radiologic evidence of progression. The Kaplan–Meier method was used to determine survival for all patients. Overall survival (OS) was defined as the time from pathologic diagnosis to the date of death or the most recent encounter with our health system, and progression-free survival (PFS) was defined as the time from pathologic diagnosis to the date of first progression or the most recent encounter with our health system. Kaplan-Meier survival analysis and was performed using GraphPad Prism (version 10.1.2 for Windows, GraphPad Software, Boston, MA, USA, https://www.graphpad.com/).  The institutional review board (IRB) of the Johns Hopkins Hospital approved this retrospective clinical data extraction and analysis.

## Results

We herein describe the clinicopathologic features of ten patients with *CIC-*rearranged sarcomas (Table 1).

**Table 1:** Clinicopathologic features of ten patients with *CIC*-rearranged undifferentiated small round cell sarcomas

Patient	Age (years), sex	Primary tumor location, size (cm)*	IHC findings	Method of *CIC* rearrangement identification	Systemic treatment	Surgery	Radiation therapy	Time from initial diagnosis to death or most recent follow-up
1	33, F	R anterior chest wall 3.5	Positive: CD99, ETV4, CAM5.2 Negative: NKX2.2, WT1, pancytokeratin, EMA, S100, SOX10, synaptophysin, CK20, CD34, CD45, desmin	FISH: positive for *CIC* rearrangement	VDC/IE	R1 excision R0 re-excision	No	NED, 24 months
2	10, M	L scalp 4.5	Positive: CD99, WT1 Negative: EMA, desmin, S100, TdT, OCT4, PLAP, pancytokeratin, NSE, synaptophysin, SOX10, CD30, CD31, CD34, CD68, MSA, MOC-31, BCOR, NKX2.2, ALK	Johns Hopkins Fusion Panel (Nanostring): negative for *CIC::DUX4* FISH: positive for *CIC* rearrangement	None	Initial excisional biopsy R0 re-excision	No	NED, 28 months
3	17, M	L gluteus maximus 18.3	Positive: CD99, O13, TLE1, ERG, CD68, EMA; retained BAF47 and INI1 Negative: AE1, AE3, S100, desmin, SMA, myogenin, HMB45, CD3, CD20, TdT, keratin	FISH: positive for *CIC* rearrangement	VDC/IE	R0 excision	Yes	DOD, 18 months
4	31, F	R lower extremity 3.9	Positive: CD56, CD99, S100 (focal) Negative: CK7, CK8/18, AE1/AE3, EMA, CD34, desmin	FISH: positive for *CIC* rearrangement	VDC/IE, IT Pazopanib Lenvatinib Trabectedin	R0 excision RLL wedge resection of lung metastases	Yes	DOD, 21 months
5	15, F	R dorsal foot 3	Positive: CD99, CAM5.2, MNF116, CD31, TLE1, NKX2.2; retained INI1 Negative: S100, HMB45, melan A, MSA, desmin, myogenin, pancytokeratin, CD34, factor 8, EMA, CD45, tyrosinase, CD163	NGS: *CIC::DUX4* fusion	None	R1 excision	Yes	NED, 52 months
6	43, F	R upper arm, bilateral lungs 8.8	Positive: TLE1, S100, cytokeratin, AE1/AE3 Negative: EMA, CAM5.2, desmin	Targeted NGS RNA sequencing: *CIC::DUX4* fusion	Ifosfamide + doxorubicin + investigational agent IT VAC	Biopsy only	Yes	DOD, 11 months
7	21, M	R posterior calf, bilateral lungs 15.6	Positive: BCOR; retained INI1 Negative: TdT, AE1/AE3, CD34, S100, myogenin, CD99	NGS: *CIC::DUX4* fusion	VDC/IE VIT Gemcitabine + docetaxel	R2 excision R0 re-excision	Yes	DOD, 21 months
8	19, M	L upper arm, bilateral lungs 3.8	Positive: WT1 (nuclear), DUX4 (nuclear), CD99 (patchy) Negative: CD30, CD34, CD45, SOX10, AE1/AE3, desmin, S100, SMA	Johns Hopkins Fusion Panel (Nanostring): negative	VDC/IE VIT	R0 excision Thoracotomy	Yes	DOD, 17 months
9	67, M	L cervical chain lymph node 2.0	Positive: p16, CD56, CD99, WT1, TLE1; retained INI1 and SMARCA4 Negative: AE1/AE3, Cam 5.2, EMA, CK7, p63, TTF1, GATA3, synaptophysin, GFAP, e-cadherin, S100, SOX10, SMA, desmin, CD34, CD45, CD3, CD20, CD10, CD30, CD31, CD68, CD117, PAX5, NKX2.2, BCOR, SATB2, NKX3.1, MDM2, STAT6, NUT1, ALK, mutant BRAF	Johns Hopkins Fusion Panel (Nanostring): *CIC::DUX4*	None	Excision, margins unreported	Yes	NED, 15 months
10	28, F	R breast 2.6	Positive: pancytokeratin, CD31, CD99, WT1 (nuclear), BCOR Negative: S100, CD34, desmin, ERG, NKX2.2	NGS: *CIC::DUX4* fusion	VDC/IE	R0 excision	No	NED, 54 months

*DOD *dead of disease, *F *female, *FISH *fluorescence in situ hybridization, *IE *ifosfamide, etoposide, *IHC *immunohistochemistry, *IT *irinotecan, temozolomide, *L *left, *M *male, *NED *no evidence of disease, *NGS *next-generation sequencing, *R *right, *VDC *vincristine, doxorubicin, and cyclophosphamide, *VIT *vincristine, irinotecan, and temozolomide

*size denotes the largest single dimension of the tumor at the time of diagnosis

### Patient 1

A 33-year-old female presented with a 3.5 cm painful mass within the subcutis of her right anterior ribcage for six months. She underwent a simple mass excision which showed a high-grade primitive round cell sarcoma harboring a *CIC* rearrangement, detected via fluorescence in situ hybridization (FISH), with microscopic positive margins. There was no evidence of distant metastatic disease. Six months after excision, the patient began Ewing sarcoma-directed therapy with vincristine, doxorubicin, cyclophosphamide, ifosfamide, and etoposide (VDC/IE) without interval compression, for a total of ten cycles. Wide local re-excision was performed after four cycles and revealed no evidence of tumor. At the time of data cutoff, the patient remains without evidence of locally recurrent or metastatic disease 24 months after diagnosis.

### Patient 2

**Fig. 1:**
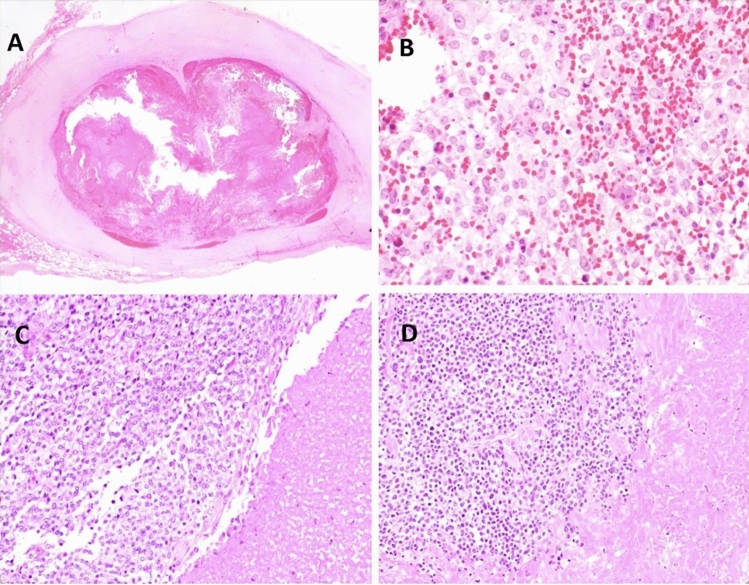
Photomicrographs of CIC-rearranged sarcomas from patients 2, 8, and 9. **A** Low-power photomicrograph from patient 2 (1.25 ×) reveals a blood-filled cystic scalp mass surrounded by a thick fibrous pseudocapsule. **B** Higher magnification demonstrates a mitotically active, high-grade primitive round cell sarcoma with lightly eosinophilic to somewhat clear cytoplasm, fine vesicular chromatin, and pinpoint nucleoli with greater nuclear pleomorphism than is generally seen in conventional Ewing sarcoma. *CIC* rearrangement was detected by FISH. **C** Histologic evaluation of patient 9 reveals a partially necrotic primitive round cell sarcoma with eosinophilic to clear cytoplasm (200 ×) which revealed a *CIC::DUX4* rearrangement. **D** Identical morphology was seen in patient 8, in which the fusion panel was negative

A 10-year-old male presented with a 4.5 cm mass on his left parietotemporal scalp that had grown slowly over approximately 17 months, with imaging reportedly suggestive of hematoma. The mass was marginally excised revealing a solid and cystic mass with internal blood products (Fig. 1A). Careful close inspection revealed a primitive high-grade round cell sarcoma with brisk mitotic activity and scant eosinophilic to somewhat clear cytoplasm (Fig. 1B). A *CIC*-rearranged sarcoma was suspected, but the Johns Hopkins Comprehensive Fusion Panel (NanoString) was negative for *CIC::DUX4*. However, subsequent FISH revealed a positive rearrangement in *CIC* supporting the histologic impression. Staging work-up revealed no evidence of metastatic disease. A wide surgical re-excision three months after the initial surgery showed no evidence of residual tumor. Chemotherapy was recommended but declined by the patient’s family. Following pathology consultation, further care was not provided at JHH, but a review of available health records indicates no evidence of locally recurrent or metastatic disease 28 months after initial diagnosis.

### Patient 3

A 17-year-old male presented with three months of left buttock pain that radiated down his leg, and MRI revealed an 18.3 cm partially necrotic left buttock mass. Biopsy revealed an undifferentiated round cell sarcoma, with a *CIC* rearrangement identified by FISH. Staging evaluation revealed enlargement of two external iliac lymph nodes (up to 1.5 cm) with mild FDG avidity, but no other evidence of metastatic disease. Lymph node biopsy was not performed. He was treated with three cycles of P6-dosed (Kushner et al. 1995), non-interval-compressed VDC before transferring care to JHH, at which time, FDG-PET revealed decreased avidity consistent with treatment response. Chemotherapy was continued with VDC/IE administered according to Children's Oncology Group (COG) protocol AEWS0031 dose and schedule, with neoadjuvant intensity-modulated radiation therapy (IMRT, 45 Gy, 25 fractions), radical resection, and the remainder of cycles administered adjuvantly. Pathology revealed 60% necrosis and negative margins. End-of-therapy CT imaging revealed two new bilateral 2 mm pulmonary nodules, which three months later had increased in size and number. The patient and their family sought care at other institutions, and limited information on subsequent treatment courses was available. The patient died 4.5 months after his last documented encounter at JHH, 18 months after initial diagnosis.

### Patient 4

A 31-year-old female presented with two weeks of a painless mass on her right anterolateral leg, and MRI revealed a 3.9 cm mass within the right tibialis anterior muscle. Staging evaluation did not reveal any clinically detectable metastatic disease. Following wide excision with negative margins, pathologic examination identified a high-grade sarcoma with *CIC* gene rearrangement identified by FISH. She was treated with four cycles of VDC and IE before MRI suggested locally recurrent disease. She received two further cycles of VDC/IE before a new finding of a right lower lobe pulmonary nodule was identified. A chemotherapy regimen consisting of irinotecan and temozolomide, for two cycles, then resulted in further progression of pulmonary metastatic disease. She underwent a right lower lobe wedge resection with the removal of three pulmonary nodules, adjuvant pazopanib for three months, and then lenvatinib for five months until she again suffered local and metastatic recurrence, with new lung and brain nodules, and local soft tissue and bony lesions in the right lower extremity. Further therapy was limited by rapidly progressive lung disease, and she died 21 months after the initial diagnosis.

### Patient 5

A 15-year-old female presented with a 3 cm mass over the dorsum of the right foot, present for at least six years by her report. The mass was initially believed to be a lipoma or cyst but recently became painful and exhibited rapid growth. She underwent an excisional biopsy, which revealed a 1.8 cm, high-grade undifferentiated round cell sarcoma with *CIC::DUX4* fusion by next-generation sequencing, with positive margins. Staging studies did not reveal any detectable distant metastatic disease. Adjuvant treatment with chemotherapy, local radiation, and surgical re-excision with wide margins was recommended, but the patient and her family declined chemotherapy and surgical re-excision. She completed radiation therapy (IMRT, 50 Gy, 25 fractions). Currently, she is more than 52 months from the initial diagnosis without evidence of local or distant metastatic recurrence on continued surveillance imaging.

### Patient 6

A 43-year-old female presented with a right upper anterior arm mass associated with two months of achiness and swelling, an associated history of trauma, and an initial ultrasound evaluation suggestive of hematoma. Following a period of rapid growth, MRI revealed an 8.8 cm mass within the right biceps muscle, and staging chest CT revealed multiple bilateral pulmonary nodules measuring up to 1.4 cm. Core biopsies diagnosed a small round blue cell tumor with extensive necrosis, and subsequent NGS analysis detected a *CIC::DUX4* fusion. The patient enrolled in a clinical trial using a backbone of ifosfamide and doxorubicin followed by local radiation treatment to the primary tumor (50 Gy, 25 fractions). There was progression of a pulmonary nodule which biopsy confirmed as metastatic disease. Second-line therapy was administered with temozolomide and irinotecan, resulting in further pulmonary disease progression and a new enlarging right axillary lymph node. Third-line therapy was administered with vincristine, cyclophosphamide, and dactinomycin for one cycle, but she experienced further progression and died approximately one month later, 11 months after initial diagnosis.

### Patient 7

A 21-year-old male presented with two months of right lower leg swelling, pain, and an enlarging mass, initially believed to be a hematoma. A core needle biopsy revealed a high-grade sarcoma with positivity for BCOR by immunohistochemistry raising the possibility of a *BCOR*-altered sarcoma. Subsequent staging studies revealed a 15.6 cm mass in the deep musculature of the right posterior calf, multiple bilateral FDG-avid pulmonary metastases measuring up to 1.2 cm, and multiple FDG-avid right popliteal and right inguinal lymph nodes. Histologic evaluation revealed a high-grade primitive round cell sarcoma, and subsequent NGS revealed a *CIC::DUX4* gene fusion. The patient was treated with Ewing sarcoma-directed therapy per COG AEWS0031. He received a total of 14 cycles of interval compressed vincristine, doxorubicin, and cyclophosphamide (VDC) alternating with ifosfamide and etoposide (IE) with wide local resection after six cycles. Pathologic examination demonstrated negative margins and 70% tumor necrosis. Postoperative radiation therapy was administered (IMRT, 55.8 Gy, 31 fractions, right calf; IMRT, 55.8 Gy, 31 fractions, right groin; IMRT, 15 Gy, 10 fractions, whole lung). The patient remained disease-free for five months before a chest CT revealed multiple left-sided pulmonary nodules and pleural effusion. He began salvage therapy with vincristine, irinotecan, and temozolomide, achieving an initial partial response of his lung nodules lasting only four cycles. Following interval growth of pulmonary metastases, the disease burden was refractory to gemcitabine and docetaxel. No further systemic cancer-directed therapy was administered, and he died from complications of metastatic lung disease three months later, 21 months after initial diagnosis.

### Patient 8

A 19-year-old male presented with an enlarging mass on his left upper arm, and MRI revealed a 3.8 cm mass within the subcutaneous tissue. Biopsy revealed a primitive round cell sarcoma with positive nuclear expression of DUX4 and WT1, suggestive of a *CIC-*rearranged sarcoma. Staging studies revealed scattered FDG-avid bilateral sub-centimeter pulmonary nodules. The patient was treated with cycles of interval compressed neoadjuvant VDC/IE, resection of the primary tumor, and additional adjuvant VDC/ IE. Pathologic examination of the tumor revealed 90% necrosis and negative margins. Progression of pulmonary metastases was noted toward the end of upfront therapy and he was treated with whole lung radiation (IMRT, 15 Gy, 10 fractions with 40 Gy boost). There was continued growth of these lesions, with the development of new pleural metastases and pleural effusion five months after completion of radiation therapy. He underwent left thoracotomy for left upper lobe wedge resection and resection of pleural metastatic disease. In-house fusion panel testing  did not detect a fusion in *CIC::DUX4*; however, the morphology and immunophenotype bore a strong resemblance to other cases in this series (Fig. 1D) in which *CIC* rearrangement was confirmed. FISH or targeted NGS was not performed on this patient’s tumor. One cycle of irinotecan, temozolomide, and vincristine (VIT) was administered, but the patient experienced rapidly progressive, symptomatic metastatic lung disease and died approximately one month later, 17 months after initial diagnosis.

### Patient 9

A 67-year-old male presented with one month of an enlarging left neck mass and CT revealed a 1.4 cm soft tissue mass abutting the left tongue base and a 2.2 cm left lower cervical chain lymph node, suggesting locoregional spread. The patient underwent tonsillectomy, tongue tissue biopsy, and excisional biopsy of three lymph nodes. All tissue was benign except for one deep cervical lymph node, which demonstrated a high-grade round cell sarcoma (Fig. 1C). In-house fusion panel testing detected a *CIC::DUX4* fusion. The patient received adjuvant radiation to the left tonsillar and neck regions. The patient remains without evidence of recurrent or metastatic disease 15 months after initial diagnosis.

### Patient 10

A 28-year-old female presented with a right breast mass with associated pain and bruising, measured to be 2.6 cm via ultrasound. Excisional biopsy was performed, and pathology revealed a small round blue cell tumor with *CIC::DUX4* fusion confirmed through FoundationOne NGS. The patient then underwent a right-sided simple mastectomy which achieved negative margins and identified one intramammary lymph node positive for disease. She completed ten cycles of adjuvant VDC/IE. Surveillance imaging revealed multiple enhancing hepatic lesions, suggestive of metastatic disease, but a biopsy revealed focal nodular hyperplasia and no evidence of malignancy. The patient remains without evidence of recurrent or metastatic disease 54 months since the initial diagnosis.

### Cohort summary

**Fig. 2:**
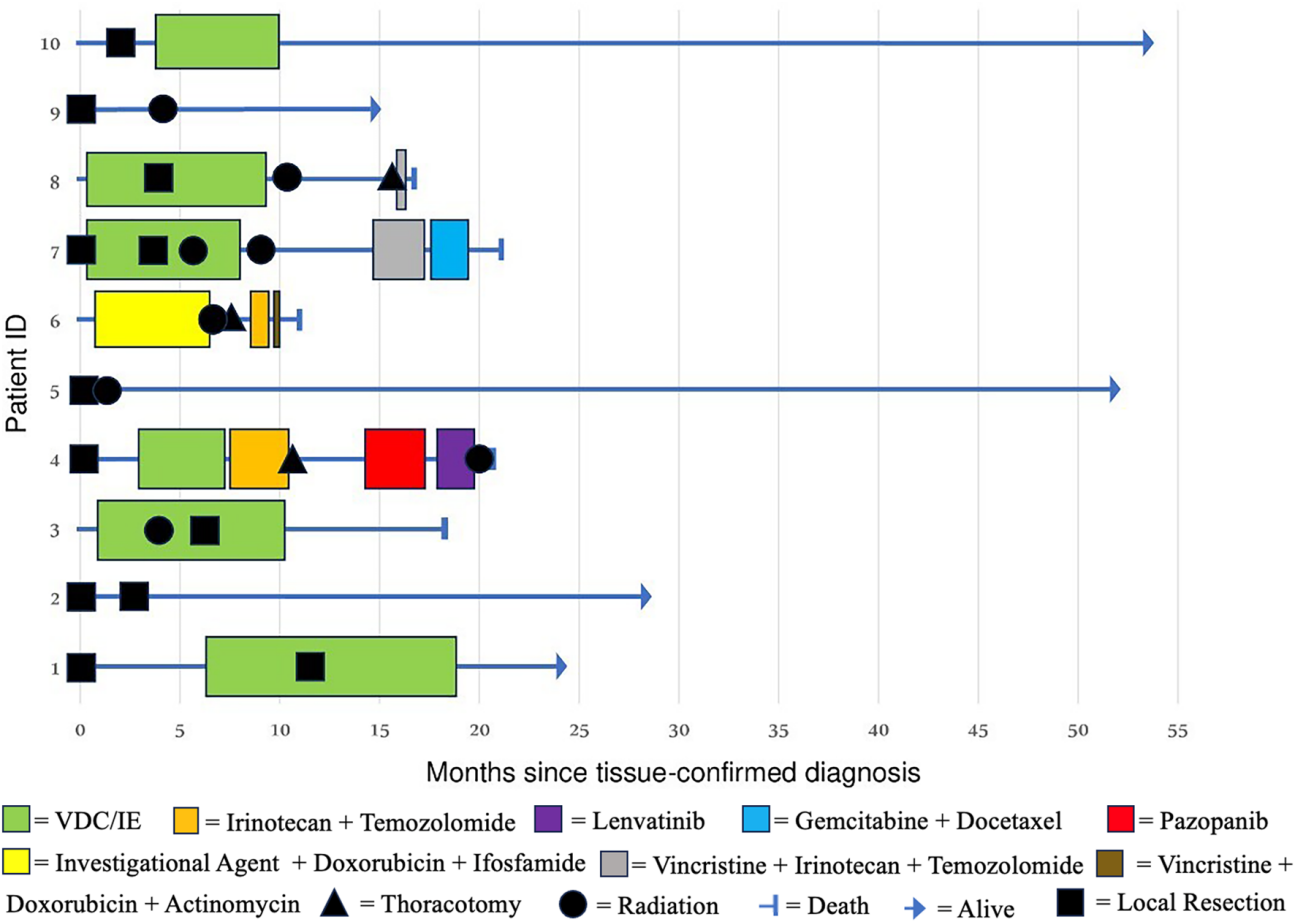
Swimmer’s plot depicting the treatment and clinical course of ten patients diagnosed with *CIC*-rearranged sarcoma

**Fig. 3:**
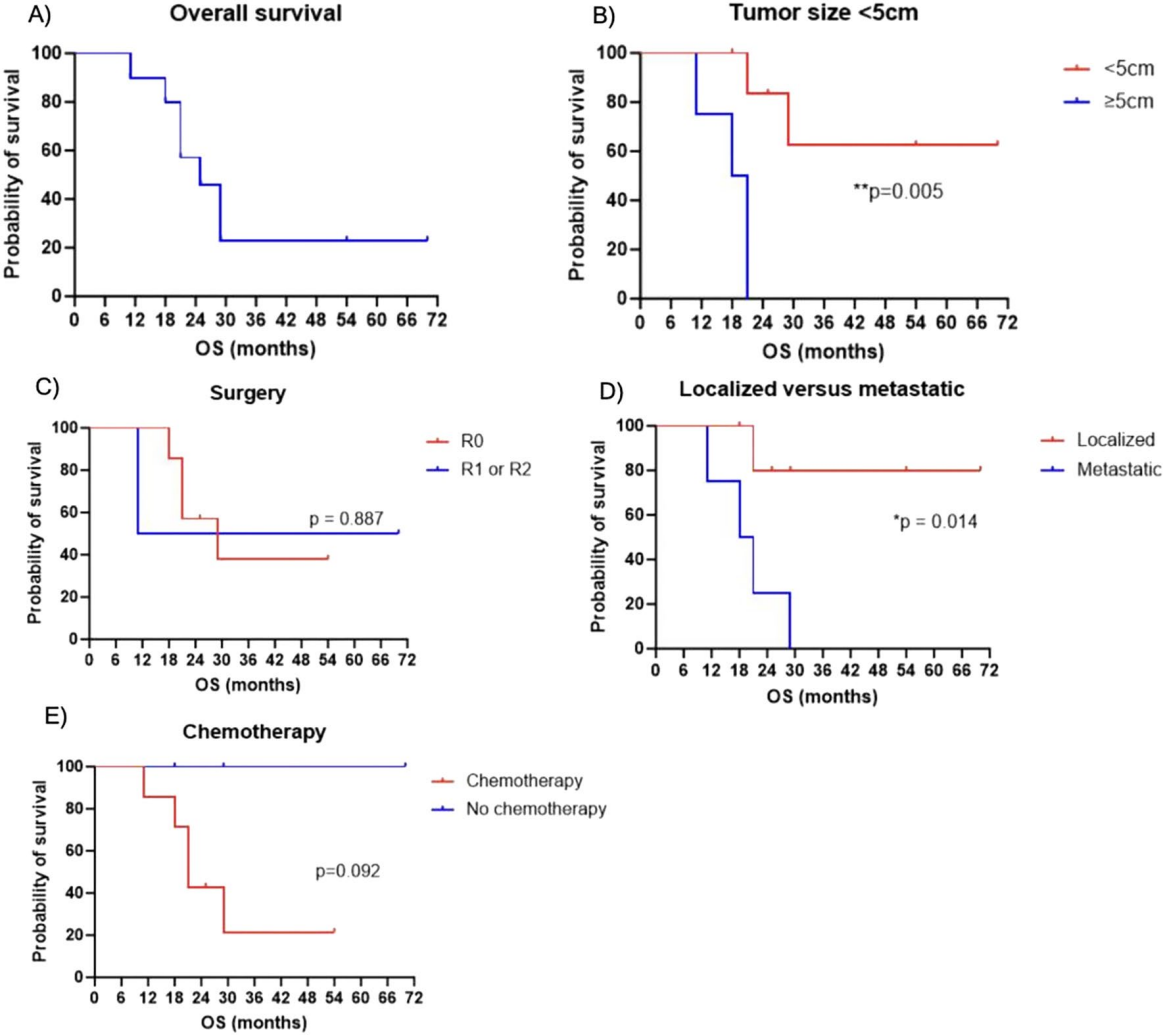
Kaplan–Meier plots depicting overall survival (months) of ten patients diagnosed with *CIC*-rearranged sarcoma. Overall survival is represented for the following: **A** the whole cohort (*n* = 10); **B** primary tumor size ≥ 5 cm or < 5 cm at diagnosis; **C** best surgical resection margins achieved for resection of the primary tumor; **D** localized (including one locoregional patient) versus metastatic disease at diagnosis; and **E** treatment with any systemic chemotherapy or not. *p < 0.05, **p < 0.01

All tumors originated from soft tissue, with locations including the breast (*n* = 1), abdominal wall (*n* = 1), scalp (*n* = 1), gluteus maximus (*n* = 1), neck (*n* = 1), lower extremity (*n* = 3), and upper extremity (*n* = 2) (Table 1). Mean overall survival for our cohort was 26.1 months and mean progression-free survival was 21.8 months from diagnosis (Table 2). (Of the seven patients who presented with localized disease, two progressed to develop metastatic disease. One patient experienced local recurrence 5.3 months after diagnosis, and the other developed distant pulmonary metastases 10 months after diagnosis (Fig. 2). These patients both died from disease, with a mean OS of 19.3 months. Patients presenting with metastatic disease at diagnosis had a mean PFS of 9.7 months and a mean OS of 16.3 months (Fig. 3).

**Table 2:** Cohort summary statistics with basic patient demographics, presenting features, and survival outcomes among ten patients with *CIC*-rearranged sarcoma

Cohort summary				
Mean age	Patient sex (M/F)	Presentation (localized /metastatic)	Overall survival (months)	Progression-free survival (months)
28.2 (range: 10–67)	5 M/5 F	7 Localized/3 Metastatic	26.1 (95% CI [16.823–35.377])	21.8 (95% CI [10.600–32.960]

Within our institutional cohort, three patients achieved long-term survival without systemic therapy. These three patients had localized disease at diagnosis. Of these, two had complete excisions with negative margins, but the third had an incomplete resection that resulted in gross residual disease. Two, including the patient with R2 resection, were treated with postoperative radiation. Additionally, all three patients had a primary sarcoma <5 cm in size. In our cohort, we observed that patients with a tumor size <5 cm experienced a significant survival advantage compared with patients presenting with primary tumors >5 cm at diagnosis (Fig. 3, Table 3).  Patients with metastatic disease had inferior survival outcomes compared to patients presenting with localized disease.

**Table 3:** Mantel–Cox log-rank test of outcomes of ten patients *with CIC*-rearranged sarcoma stratified by tumor size, extent of surgery, metastasis at diagnosis, and exposure to chemotherapy. * indicates hazard ratio calculated using Mantel–Haenszel method

	*p* value	Hazard ratio	95% CI of hazard ratio
Tumor size < 5 cm	0.005	0.154	0.023 to 1.016
Surgery	0.887	0.858	0.086 to 8.545
Metastasis at diagnosis	0.014	8.864	1.334 to 58.89
Chemotherapy	0.092	5.299*	0.763 to 36.82

## Discussion

*CIC*-rearranged sarcomas represent a rare type of small round cell sarcoma typically associated with an aggressive clinical course. Research on these sarcomas is limited due to the disease rarity and its relatively recent recognition as an entity distinct from Ewing sarcoma, with case series and retrospective studies informing the majority of literature on this topic. To aid in diagnostic distinction, judicious application of molecular genetics, including comprehensive fusion panels, should be obtained for all patients with unclassified small round cell sarcomas. If a *CIC-*rearranged tumor is suspected and the fusion panel does not detect a fusion, further evaluation should be pursued via immunohistochemistry, FISH, and/or NGS to provide the most accurate molecular classification when possible. There have been no known clinical or prospective studies on the optimal management of *CIC*-rearranged sarcomas, and further research is essential to improve clinical decision-making for affected patients. Retrospective reviews of *CIC*-rearranged sarcomas have highlighted the aggressive nature of these tumors, specifically their chemo-resistant nature, high frequency of metastasis, and rapid development of treatment refractoriness (Connolly et al. 2022). Our study broadens the spectrum of reported clinical outcomes of these patients, including three patients managed without systemic chemotherapy who achieved long-term survival, highlighting a departure from the typical soft tissue sarcoma treatment paradigm of systemic chemotherapy with local surgical and/or radiotherapeutic control.

Within our institutional series of patients with *CIC*-rearranged sarcomas, seven out of ten patients received intensive systemic therapy with an Ewing sarcoma-directed regimen (VDC/IE) or a soft tissue sarcoma-directed regimen (doxorubicin/ifosfamide). Of these patients, three presented with metastatic disease at diagnosis; five are deceased (overall survival range: 11–22 months); the remaining two surviving patients presented with localized disease, and at the time of data cutoff are without evidence of disease. Of the deceased patients, four (of five) demonstrated initial disease response, but eventually developed rapidly progressive and refractory metastatic disease. In comparison to the seven patients who received traditional Ewing sarcoma-like treatments, three (of ten) patients analyzed in our cohort did not receive systemic chemotherapy and are alive without evidence of disease at the time of data cutoff (overall survival range: 15–67 months from diagnosis). These patients had no evidence of metastatic disease at diagnosis and all underwent local resection, with two patients treated with local post-operative radiation.

It is worth noting the immunohistochemical staining positivity for BCOR in patients 7 and 10. While BCOR-altered sarcomas are recognized as a distinct entity within USRCSs , BCOR positivity on immunohistochemistry cannot be used to infer the absence or presence of a specific molecular rearrangement. BCOR positivity by immunohistochemistry is reported as a nonspecific finding shared across multiple sarcoma subtypes, and while initially thought to be a feature more unique to BCOR-rearranged sarcomas, its presence in a variety of tumors makes it a sensitive but nonspecific diagnostic finding (Pan et al. 2023; Astolfi et al. 2019; Antonescu et al. 2017).

While the majority of our patients demonstrated aggressive clinical courses consistent with those described in the literature, we note several unique features in our cohort, and a spectrum of clinical outcomes which, to our knowledge, have not been previously reported. Contrary to expectations, three patients experienced prolonged disease-free survival without intensive systemic treatment (patients 2, 5, and 9). Additionally, two patients reported the appearance of a mass at the primary tumor site years before their tissue diagnosis (patients 2 and 5). In both cases, the mass was reportedly present and unchanged for years before demonstrating rapid growth and/or development of symptoms (e.g., pain), prompting tissue biopsy and diagnosis. The fact that several patients with localized disease achieved prolonged disease remission without systemic treatment suggests that their behavior may be driven more by their individual biology (including secondary molecular alterations) and not necessarily the treatments administered. This idea is further supported by the relationship between patient outcome and primary tumor size, as most patients in this cohort with tumors less than 5 cm in size achieved long-term survival and those with tumors greater than 5 cm all died. If true, this idea would be a departure from the traditional Ewing sarcoma treatment paradigm by which most patients with *CIC*-rearranged tumors are treated.

This study is limited by a small sample size and retrospective design. For some patients who received part of their care at outside institutions, our summaries are based on an electronic review of records from outside institutions. Despite these limitations, these patients’ experiences provide a valuable addition to the published literature on this rare cancer, highlighting the spectrum of clinical outcomes for these patients. Most importantly, this study emphasizes the need for prompt pathologist recognition and judicious application of molecular genetics (such as FISH, and/or targeted NGS) and future prospective and/or randomized studies to better understand the biology and what factors influence behavior to improve future outcomes.
